# Flow Behaviors of Polymer Solution in a Lid-Driven Cavity

**DOI:** 10.3390/polym14122330

**Published:** 2022-06-09

**Authors:** Cuong Mai Bui, Anh-Ngoc Tran Ho, Xuan Bao Nguyen

**Affiliations:** The University of Danang—University of Technology and Education, 48 Cao Thang, Da Nang 550000, Vietnam

**Keywords:** non-Newtonian fluid, yield stress, polymer rheology, cavity flow

## Abstract

In this work, a numerical study of polymer flow behaviors in a lid-driven cavity, which is inspired by the coating process, at a broad range of Oldroyd numbers (0≤Od≤50), is carried out. The Reynolds number is height-based and kept at Re=0.001. The fluid investigated is of Carbopol gel possessing yield stress and shear-thinning properties. To express rheological characteristics, the Herschel–Bulkley model cooperated with Papanastasiou’s regularization scheme is utilized. Results show that the polymer flow characteristics, i.e., velocity, viscosity, and vortex distributions, are considerably influenced by viscoplastic behaviors. Additionally, there exist solid-like regions which can be of either moving rigid or static dead types in the flow patterns; they become greater and tend to merge together to construct larger ones when Od increases. Furthermore, various polymer flow aspects in different cavity configurations are discussed and analyzed; the cavity width/aspect ratio and skewed angle are found to have significant impacts on the vortex structures and the formation of solid-like regions. Moreover, results for the critical aspect ratio at which the static dead zone is broken into two parts and the characteristic height of this zone are also reported in detail.

## 1. Introduction

The lid-driven cavity flow has been widely realized for benchmarking computational fluid dynamics (CFD) approaches not only due to its simple geometric setup but also since it sufficiently exhibits basic hydrodynamic features. Additionally, this problem has various important engineering applications such as solar collector [1], fluid mixing [2], and polymer coating processes [3]. In fact, the flow behaviors inside a lid-driven cavity was extensively studied using analytical, experimental, and numerical techniques; Shankar and Deshpande [4] provided a comprehensive review on this problem. To mention a few, Ghia et al. [5] proposed a flow modeling approach with the multigrid method and then investigated the shear-driven flow in a square cavity. Various flow features, e.g., velocity field, vorticity distribution, and flow streamlines, at Re≤104 were revealed. The results for Re≤103 were then compared with those obtained by experimental works of Mochizuki et al. [6]; good agreements were observed in terms of flow field pattern and velocity distribution. As indicated, there existed three vortices, a large one near the cavity’s center (i.e., primary vortex) and two much smaller at its corners (i.e., secondary vortices). The location and size of these vortices were stated to depend on Reynolds number. More recently, Xu et al. [7] and Khorasanizade and Sousa [8] carried out the flow analysis at, respectively, Re = 10–1000 and Re = 400–3200 using the newly developed Smooth Particle Hydrodynamics (SPH) method.

It is worth reminding that the fluids investigated in the aforementioned studies are of a Newtonian type whose viscosity does not vary during the flowing. This assumption is supposed to not be suitable for many engineering flows in which the fluids can possess rheologically complex properties such as shear-dependence, yield stress, viscoelasticity, or thixotropy. Examples of non-Newtonian materials can range from mineral suspensions [9,10], clay suspensions [11,12], and lava [13,14] to crude oil [15,16,17], melting metal [18,19], printing ink [20,21], painting [22], polymer [23,24,25,26,27,28], or even human blood [29,30]. To deal with the lid-driven cavity flow problem of these fluids, computational approaches were mainly adopted in previous works. Amongst them, the Bingham type was the most focused [31,32,33,34,35,36]. For such fluid, the flowing occurs provided that the applied shear stress exceeds the yield stress of the material; moreover, the correlation between the shear stress and deformation is assumed to be linear during the flowing. Compared to a Newtonian fluid, the flow structures of a Bingham one were found to be more complex with the development of unyielded zones, which were solid-like, in the flow pattern when Bn>0. In these works, artificial fluids with rheological properties added was considered. Recently, Hoang–Trong et al. [37] provided information of the flow morphology of real Bingham fluids, i.e., kaolinite suspensions, at Re = 100–1000 in rectangular lid-driven cavities. As indicated, the unyielded zones became greater with the increase in the kaolinite concentration. Moreover, the effects of aspect ratio on the flow morphology was determined to be significant. For different non-Newtonian types, Pakdel and his team published a series of experimental results for polymer–oil solutions, which was assumed to possess a constant elastic viscosity, in [38,39,40]. Viscoelastic fluids were also discussed and well analyzed in both square [41,42,43] and rectangular cavities [43]. Mahmood et al. [44] and Shuguang [45] described the power-law fluid behaviors by Carreau model and performed simulations in the square cavity with, respectively, finite element and finite deference methods; results indicated that the impacts of power-law index became more significant with the larger Re.

In this work, we are aiming at exploring the flow morphology of a polymer solution in a lid-driven cavity. The gel targeted exhibits both viscoplastic (i.e., yield stress effect) and shear-thinning behaviors, making the investigation relevant to the application case of the polymer coating. Furthermore, the influences of cavity configurations on the flow structures are also examined; this would contribute to optimizing the coating process designs.

The remaining of this paper is organized as follows: Section 2 introduces the research approach, i.e., the governing equations and simulation implementation; Section 3 provides and analyzes the results obtained; and concluding remarks are given in Section 4.

## 2. Methodology

### 2.1. Governing Equations

The laws of mass and momentum conservations for a fluid flow are, respectively, expressed as

(1) ∇·u=0,

(2) $$\rho \left(\frac{\partial u}{\partial t}+u\cdot \nabla u\right)=\rho f+\nabla \cdot \sigma \;,$$

where u is the velocity vector and ρ the fluid density; σ is the total stress tensor and determined as

(3) σ=−pI+τ,

where *p* is the pressure, and I the unit tensor. Additionally, τ is the shear stress tensor; for a Newtonian liquid, it is defined as

(4) $$\tau =\mu \dot{\gamma }\;,$$

with μ being the fluid viscosity and $\dot{\gamma }$ the strain rate tensor. For a non-Newtonian liquid, τ can be generally expressed using Herschel–Bulkley (HB) model as follows [46],

(5) $$\left\{\begin{array}{cc} \tau =\left(K{\dot{\gamma }}^{n-1}+\frac{\tau_{0}}{\dot{\gamma }}\right)\dot{\gamma } & \mathrm{if}\;\tau >\tau_{0}\\ \dot{\gamma }=0 & \mathrm{if}\;\tau \leq \tau_{0}\;. \end{array}\right.$$

Here, *K* is the plastic viscosity, *n* the power index controlling the curve of $\dot{\gamma }$-τ after the fluid material being yielded, and $\tau_{0}$ is the yield stress. Furthermore, $\dot{\gamma }=\sqrt{\frac{1}{2}\dot{\gamma }:\dot{\gamma }}$ and $\tau =\sqrt{\frac{1}{2}\dot{\tau }:\dot{\tau }}$ are the strain rate magnitude and the intensity of the extra stress, respectively.

In addition, to generally characterize the viscoplastic fluid flow, Reynolds (Re) and Oldroyd (Od) numbers are, in turn, defined as follows,

(6) Re=ρu02−nHnK,

(7) $$\mathrm{Od}=\frac{\tau_{0}H^{n}}{K{u_{0}}^{n}}\;,$$

where $u_{0}$ is the incoming velocity and *H* the cavity’s height. It is worth mentioning that in this work, the flow is very slow with Re being kept at Re=0.001.

### 2.2. Fluid Materials

In this work, we use water properties to describe the Newtonian characteristics as ρ = 1000 $\mathrm{kg}/m^{3}$ and μ=0.001$\;\mathrm{Ns}/m^{2}$.

**Figure 1:**
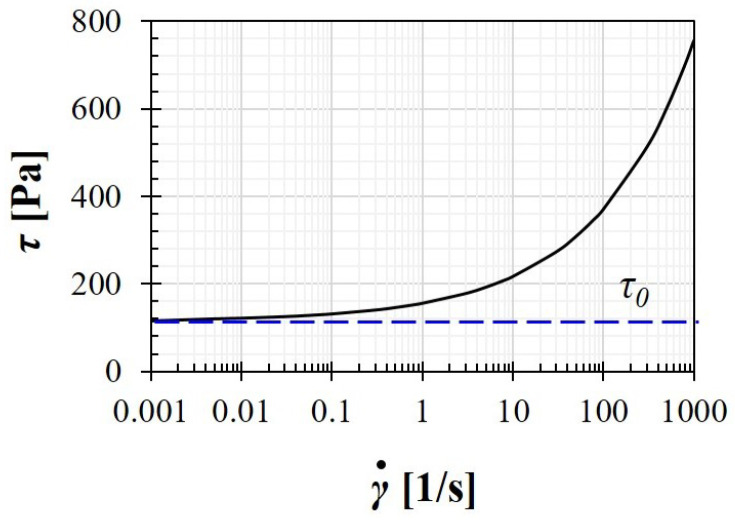
Correlation between the shear stress τ and the deformation rate $\dot{\gamma }$ during the flowing of Carpobol gel.

For the non-Newtonian material, the polymer solution prepared with Carbopol 940, which was adopted in experimental series of Ouattara et al. [26], is utilized. Specifically, its rheological properties are determined to be of K=40.4$\;\mathrm{Pa}\;s^{n}$, n=0.4, and $\tau_{0}=115$ Pa. Furthermore, the fluid density is chosen as ρ=1000$\;\mathrm{kg}/m^{3}$. The influences of the material microstructural evolution are neglected. With HB model, the flowing curve of this material can be illustrated in Figure 1.

### 2.3. Computational Approach

#### 2.3.1. Regularization Scheme

As can be seen in Equation (5), there has a discontinuous point at $\tau =\tau_{0}$, possibly leading to unexpected numerical oscillations and/or deviations. Furthermore, with this model, the deformation rate is assumed to be zero at the yield point and below it. This is unreasonable with real materials; for instance, Carbopol gel has the critical shear rate of $\dot{\gamma_{c}}=0.001$ s^−1^ showing a non-zero deformation when $\tau =\tau_{0}$ [26]. To tackle the mentioned issues, Papanastasiou’s regularization method is employed as [47]

(8) $$\tau =\left(K{\dot{\gamma }}^{n-1}+\frac{\tau_{0}\left[1-\exp\left(-m\dot{\gamma }\right)\right]}{\dot{\gamma }}\right)\dot{\gamma }\;,$$

where *m* is the regularization parameter. It is determined to ensure the flowing curve to pass the point of ($\dot{\gamma_{c}}$, $\tau_{0}$) [48,49]. In detail, it is m=3809 for the Carbopol gel adopted [49].

#### 2.3.2. Computational Implementation

**Figure 2:**
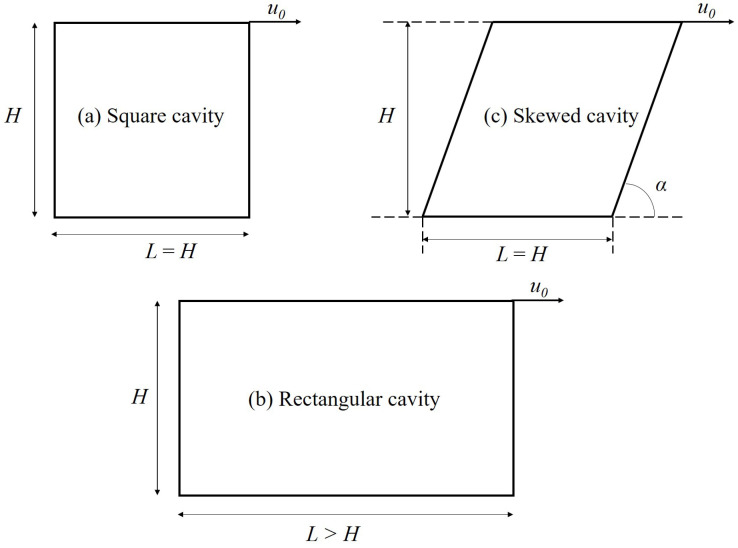
Geometry of the lid-driven cavities.

**Figure 3:**
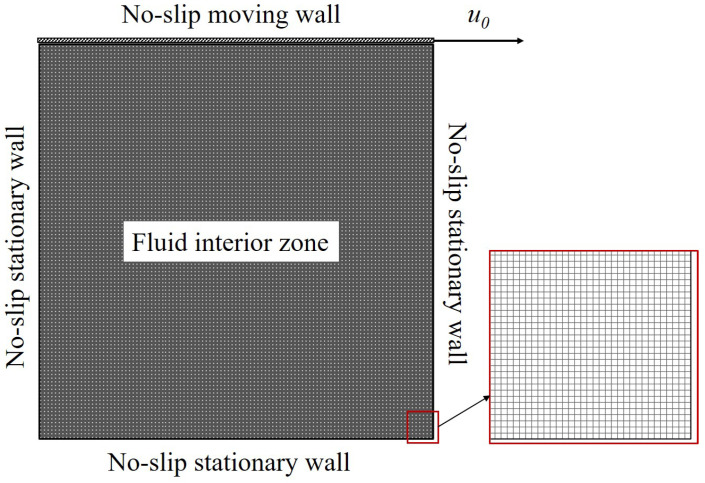
Computational mesh for a square cavity and boundary conditions.

**Figure 4:**
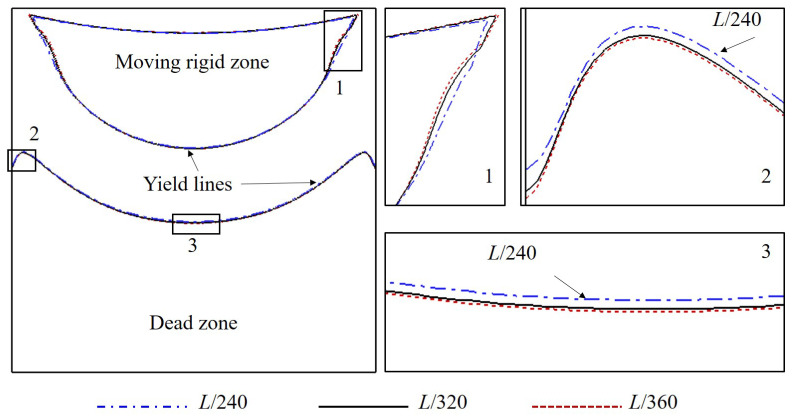
Influence of mesh refinement on the yield lines of $\dot{\gamma }=0.001$ s^−1^ in a square cavity at Od=10.

**Figure 5:**
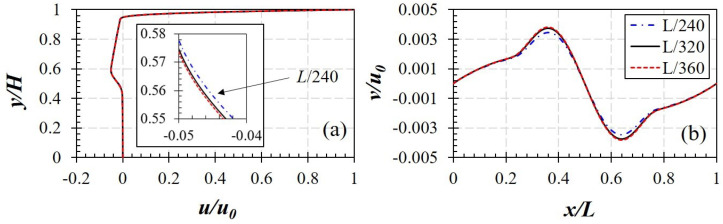
Influence of mesh refinement on velocity distribution: (**a**) *x*-velocity along the vertical centerline and (**b**) *y*-velocity along the horizontal centerline of a square cavity at Od=10.

Figure 2 shows the geometry of the problem studied in this work including the square, rectangular, and skewed cavities. Moreover, computational mesh and boundary conditions applied are provided in Figure 3. Only the top wall is moving with $u=u_{0}$; other walls are stationary. For the calculations, the domain is discretized to a system of structured mesh constituted by uniform square elements. Additionally, a mesh sensitivity study has been conducted to detect the most optimal resolution. In detail, three resolutions with different mesh element widths, i.e., Δx=L/240, Δx=L/320, and Δx=L/360, are tested. Figure 4 illustrates the influences of mesh resolution on the yield lines at Od=10; it is indicated that the deviations become insignificant with Δx≤L/320. Additionally, as can be observed in Figure 5, the velocity distributions along vertically and horizontally central lines produced by the mesh widths of Δx=L/320 and Δx=L/360 are nearly the same. It is worth mentioning that the former shows the greater computational efficiency; specifically, it takes ∼11.5 h meanwhile the mesh of Δx=L/360 requires up to ∼18 h for the solution convergence. Therefore, we select the mesh width of Δx=L/320 for all simulations. Furthermore, the calculations are performed using the Finite Volume Method (FVM); second-order schemes are used for spatial discretization.

**Figure 6:**
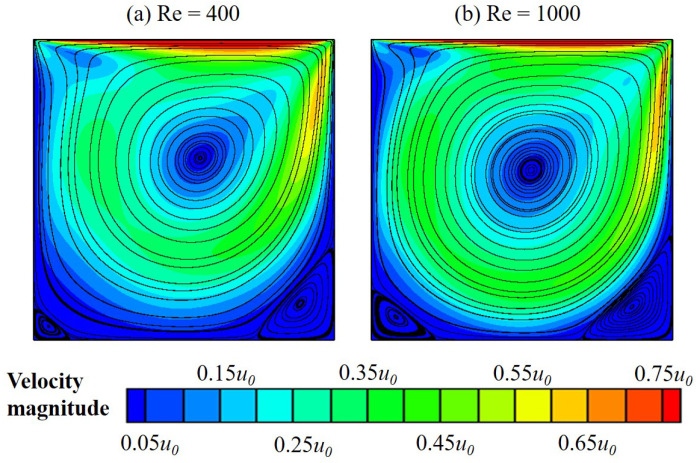
Flow streamlines of a Newtonian fluid in a square lid-driven cavity at (**a**) Re=400 and (**b**) Re=1000.

**Figure 7:**
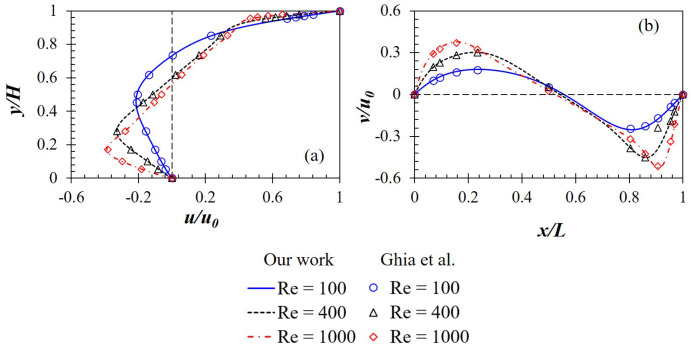
(**a**) *x*-velocity along the vertical centerline and (**b**) *y*-velocity along the horizontal centerline produced by a Newtonian fluid in a square cavity at Re = 100, 400 and 1000.

Due to the lack of experimental data for non-Newtonian fluid flow, we perform the validation with the Newtonian one. Our simulation results at Re=400 and 1000 shown in Figure 6 are compared to those experimentally obtained by Mochizuki et al. [6]. It is good to observe that ours agree very well with the experimental results; in detail, both affirm the appearance of vortices at the cavity center and the corners. In addition, results for velocity magnitude provided by our numerical approach also perfectly match those in Ghia et al. [5] at Re = 100, 400 and 1000 (see Figure 7).

## 3. Results and Discussion

### 3.1. Polymer Flow Morphology

The polymer flow structures in a square lid-driven cavity at Re=0.001 are discussed and analyzed in this part. Values of Oldroyd number are ranged in Od = 0–50. It is noted that the extreme case of Od=0 represents the Newtonian fluid flow.

**Figure 8:**
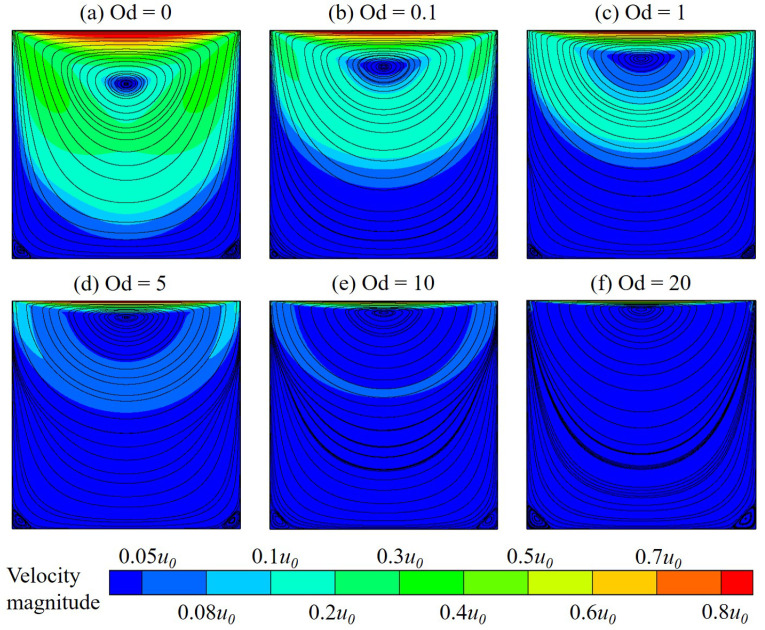
Velocity contour and flow streamlines of the polymer flow in a square lid-driven cavity at Od = 0–50.

**Figure 9:**
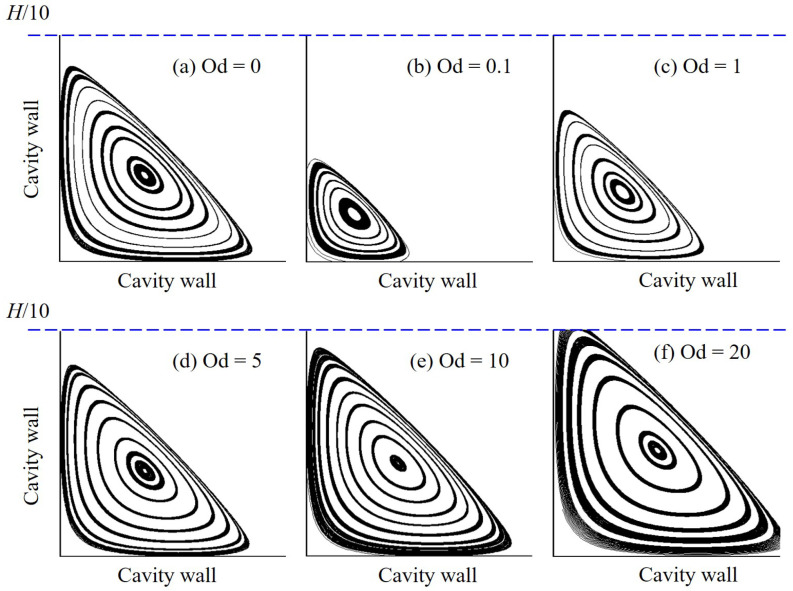
Formation of the left secondary vortex obtained by the polymer flow in a square lid-driven cavity with various values of Od.

Results for the velocity distribution and flow streamline patterns are shown in Figure 8. The variation zone of velocity is observed to narrow and get closer to the moving (top) wall when Od increases. At high Od, i.e., Od≥5, it occurs that the velocity magnitude is smaller than $0.1u_{0}$ in almost the whole cavity, indicating a very slow motion of the fluid there when the viscoplastic effect becomes profound. Similar to the Newtonian fluid flow (i.e., Od=0), there appear one primary and two secondary vortices in the flow field pattern of the polymer gel; however, their location and size differ with various values of Od. For instance, the primary vortex eye is found to move up with the increasing Od. Moreover, for the polymer flow, the larger Od, the greater size of the left secondary vortex, and the further its center is positioned from the cavity corner (see Figure 9). Due to the similar flow distribution at two corners when the inertial effect is negligibly insignificant, the right secondary vortex is nearly the same as the left one (see Figure 8); the finding observed for the latter is also reasonable for the former.

**Figure 10:**
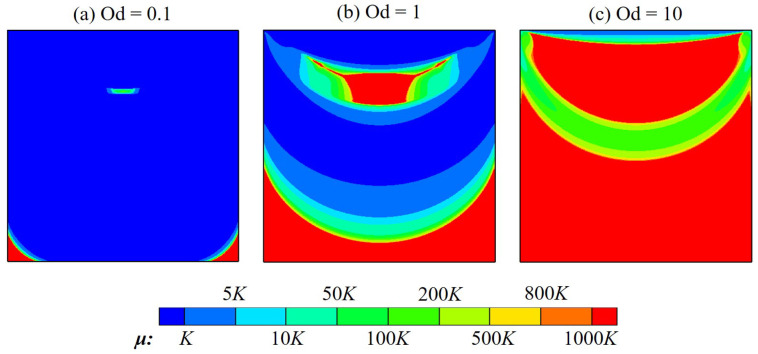
Viscosity distribution of the polymer flow in a square cavity at various Od.

**Figure 11:**
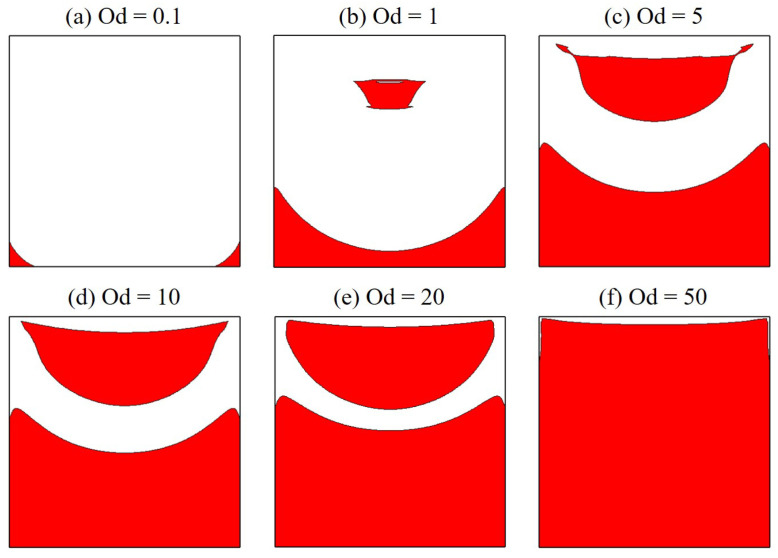
Solid-like zones ($\dot{\gamma }\leq 0.001$ s^−1^) as the red areas produced by the polymer flow in a square cavity at various Od.

Figure 10 illustrates the viscosity field at several Od. As can be seen, highly viscous areas are noticed to develop in the flow pattern; these zones significantly extend as Od is increased. Indeed, the polymer viscosity during the flowing can even be 1000 times greater than the plastic viscosity, in almost the entire cavity at Od=10. The existence of high-viscosity zones, which prohibit fluid displacement, is probably the main reason for the very slow movement of polymer flow mentioned above. Furthermore, solid-like regions can be formed inside these zones. Within them, the polymer solution is unyielded; the material deformation is very small as $\dot{\gamma }\leq 0.001$ s^−1^. Results for unyielded zones with various Od are shown in Figure 11. It is evident that the unyielded zones can be classified into two different types: the moving rigid and the dead zone firmly adhering to the bottom and sidewalls of the cavity. As Od is greater, unyielded zones considerably expand and tend to assemble to become large ones. Indeed, when Od is in the range of Od = 1–20, there still appear two types of unyielded zones clearly in the flow field pattern; however, only a very large dead zone filling the whole cavity is found at the highest value of Od investigated, i.e., Od=50. The appearance of such a large non-moving rigid sticking on cavity walls should be paid a great attention since it blocks the flow circulation and then negatively affect the operation of the lid-driven cavity scheme.

### 3.2. Cavity Configurations

In this part, effects of cavity configurations on the polymer flow characteristics, especially the formation of unyielded regions, are presented and examined. Rectangular and skewed cavities are, respectively, investigated. Moreover, regarding the mesh employed, the resolution of Δx=L/320 is continued to be realized.

#### 3.2.1. Rectangular cavity

We carry out simulations on rectangular cavities with different widths; the aspect ratio is varied in range of Λ=L/H = 0.5–4.

**Figure 12:**
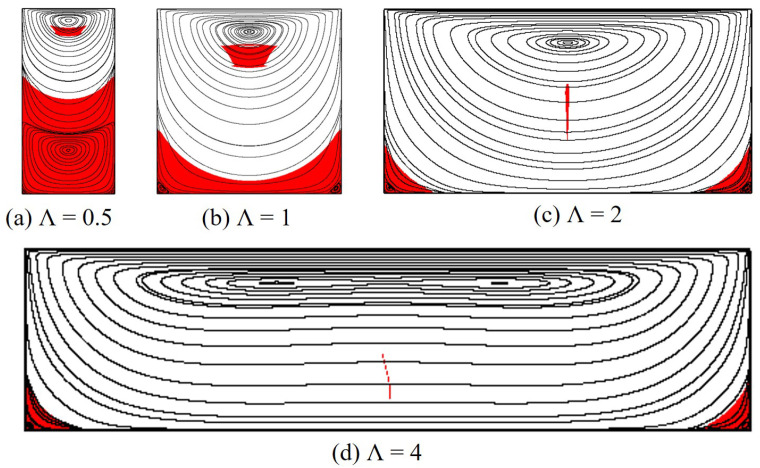
Flow morphology of the polymer gel in rectangular cavities with different aspect ratios at Od=1.

**Figure 13:**
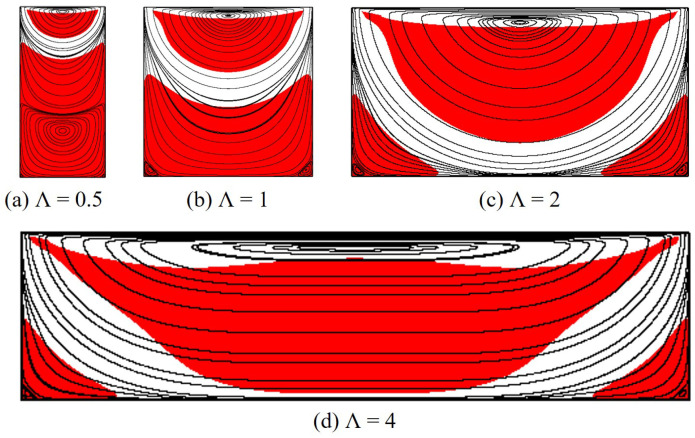
Flow morphology of the polymer gel in rectangular cavities with different aspect ratios at Od=10.

Results for flow streamlines and unyielded zones produced by the polymer flow at Od=1 and Od=10 are, respectively, presented in Figure 12 and Figure 13. It is noticeably seen that another primary vortex is formed in the lower half of the cavity with Λ=0.5 (see Figure 12a and Figure 13a); this was also observed for the Newtonian [4] and Bingham fluid flows [37]. Additionally, the primary vortex (only the upper one for Λ=0.5) is found to lengthen and shift down with the increasing aspect ratio for both Od=1 and Od=10. For the unyielded zones, at Od=1, the moving rigid region generally shrinks when the cavity is greater in width; with Λ=4, it nearly disappears. However, this trend is reversed for Od=10 at which the moving rigid considerably enlarges as Λ increases.

**Figure 14:**
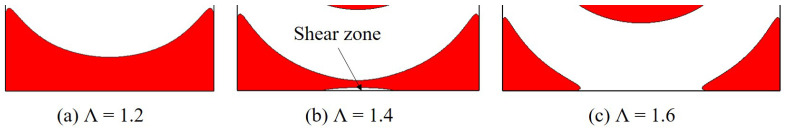
Transformation in the dead zone with various cavity widths at Od=5.

Furthermore, the dead zone dramatically varies with the cavity width. It tends to split into two smaller ones attaching to the cavity corners as the aspect ratio is large. When Λ increases, due to the polymer shear at the middle of the cavity bottom, the rigid layer there becomes thinner (see Figure 14). It is then fragmented into two parts as discussed above when the aspect ratio reaches a critical value, $\Lambda_{c}$. Table 1 reveals results of $\Lambda_{c}$ for different values of Od. As can be seen, the larger Od, the greater the aspect ratio required to divide the dead zone; for instance, at very high Od, e.g., Od=20 or 50, $\Lambda_{c}$ is found to be even larger than 4 meanwhile it is only $\Lambda_{c}=0.8$ for Od=0.1. In addition, the detailed results for the characteristic height, $H_{s}$, of the dead zone are reported in Table 2. The cavities of L=2H and L=4H are seen to provide a similar results for $H_{s}$ at all Od studied. Moreover, it is evident that $H_{s}$ is decreased with the greater cavity width at Od = 0.1–20; for Od=50, the characteristic height is remained at $H_{s}=0.99$, indicating the nearly full coverage of the dead zone in the whole cavity for a wide range of Λ.

#### 3.2.2. Skewed Cavity

The flow morphologies, i.e., formation of unyielded zones and vortex distribution, in a cavity with various skewed angles are presented and discussed. The schematic of the skewed cavity can be found in Figure 2c. The skewed angle is in the range of α = 45–120${}^{o}$; moreover, Od is kept constant at Od=1 in this part.

**Figure 15:**
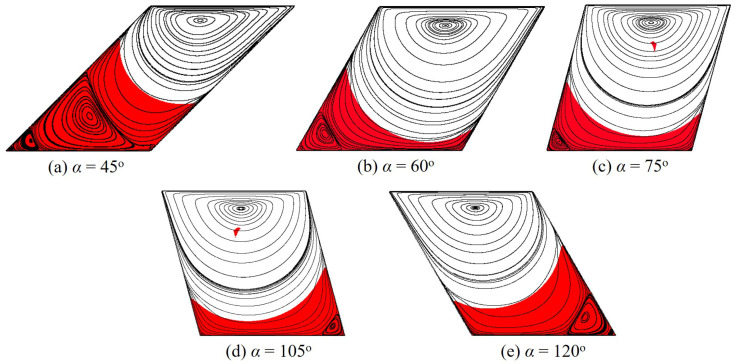
Flow morphology of the polymer gel in skewed cavities with different α at Od=1.

Figure 15 illustrates the polymer flow structures in skewed cavities with various values of α. Different from the straight cavity (i.e, $\alpha =90^{o}$), the distribution of the dead zone is asymmetrical about the horizontally central line when the sidewall tilts. In detail, with $45^{o}\leq \alpha <90^{o}$, the dead zone clinging on the left wall is higher while it is lower on the right one with $90^{o}<\alpha \leq 120^{o}$. Furthermore, the moving rigid obtained by the skewed cavities does not exist or is very small compared to that created by a straight one (see Figure 11b).

**Figure 16:**
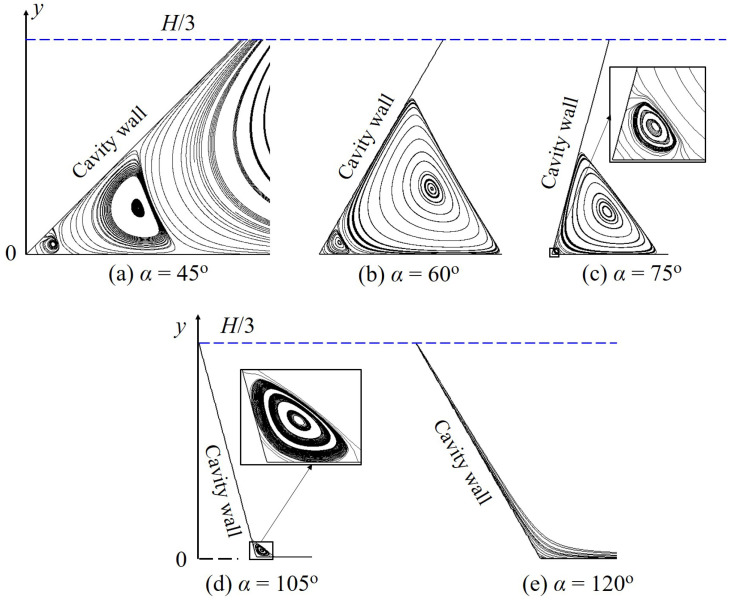
Vortex structures at the left corner produced by the polymer flow in the skewed cavities with different α at Od=1.

In addition, the vortex characteristics, i.e., quantity, location and size, is observed to be significantly affected by the skewed angle (see Figure 16). For example, at the left corner, the number of secondary vortex is reduced with the increasing α; specifically, this quantity is of three for $\alpha =45^{o}$ but decreases to one and even zero with, respectively, $\alpha =105^{o}$ and $\alpha =120^{o}$.

## 4. Concluding Remarks

The creeping lid-driven cavity flow characteristics of a polymer solution at Re=0.001 and Od = 0–50 were numerically investigated in this work. The fluid targeted simultaneously exhibited yield stress and shear-thinning features; the rheological behaviors were described by the Herschel–Bulkley model coupled with Papanastasiou’s regularization technique.

The polymer flow characteristics were determined to be greatly dependent on the viscoplastic property. In detail, when the yield stress effect was serious, i.e., Od was large, the velocity magnitude became very small inside the cavity. Moreover, the vortex distribution was varied with different Od. For instance, as Od was larger, the primary vortex shifted up; additionally, the secondary vortices became larger and moved further away from the cavity corners. Furthermore, solid-like regions including moving rigid and (static) dead zones were found to develop in the polymer flow field patterns, reducing the flowing and then the lid-driven cavity operation. These zones were significantly enlarged and tended to merge together to form larger ones when Od increased.

In addition, the formations of vortices, moving rigid regions, and dead zones were strongly affected by the cavity configuration. For the rectangular cavity, as the aspect ratio increased to a critical value, the dead zone broke into smaller ones sticking on two bottom corners; $\Lambda_{c}$ was found to be greater with the increasing Od. Detailed results for $\Lambda_{c}$ and the characteristic height of the dead zone are also provided. Furthermore, the skewed angle also had considerable impacts on the polymer flow morphology, especially the quantity, location, and size of vortices.

## Figures and Tables

**Table 1:** Results for critical aspect ratio $\Lambda_{c}$.

Od	0.1	1	5	10	20	50
$\Lambda_{c}$	0.8	1.2	1.6	1.8	>4	>4

**Table 2:** Results for characteristic height $H_{s}/H$ of the dead zone with different cavity widths.

		Λ	0.5	1	2	4
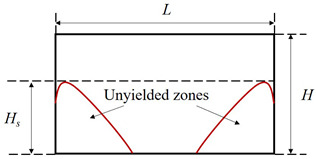		Od=0.1	0.46	0.12	0.11	0.11
		Od=1	0.63	0.34	0.26	0.26
		Od=5	0.75	0.54	0.42	0.4
		Od=10	0.79	0.6	0.49	0.48
		Od=20	0.82	0.65	0.56	0.56
		Od=50	0.99	0.99	0.99	0.99

## Data Availability

Not applicable.
